# Effects of anti-*H. pylori* triple therapy and a probiotic complex on intestinal microbiota in duodenal ulcer

**DOI:** 10.1038/s41598-019-49415-3

**Published:** 2019-09-06

**Authors:** Lili Wu, Zikai Wang, Gang Sun, Lihua Peng, Zhongsheng Lu, Bin Yan, Kun Huang, Yunsheng Yang

**Affiliations:** 1 Department of Gastroenterology, First Medical Center, Chinese People’s Liberation Army General Hospital, Beijing, China; 2 Department of Gastroenterology, Second Medical Center, Chinese People’s Liberation Army General Hospital, Beijing, China; 3 Department of Gastroenterology, Civil Aviation General Hospital, Beijing, China

**Keywords:** Gastroenteritis, Duodenal ulcers

## Abstract

This study aimed to investigate the intestinal microbiota in duodenal ulcer (DU) patients, effects of proton pump inhibitors,clarithromycin and amoxicillin, PCA) for *Helicobacter pylori* (*H*. *pylori*) and *Bacillus subtilis* and *Enterococcus faecium* (BSEF) on intestinal microbiota. DU patients were randomly assigned to receive either PCA (group TT) or PCA plus BSEF(group TP). The fecal microbiome was conducted using high throughput 16S rDNA gene and internal transcribed spacer sequencings. The diversity and abundance of intestinal bacteria in the DU were significantly lower than health check control (HC) group. In the TT group, the abundance and diversity of both intestinal bacteria and fungi decreased after PCA treatment, compared with those before treatment, whereas in the TP group no obvious changes were observed. In the TT group at all the time points, both the intestinal bacteria and fungi were different from those in the HC group. However, in the TP group, at 10w the bacterial flora abundance was close to that in the HC group. The results indicate that anti- *H*. *pylori* treatment induced significant decrease in the diversity of intestinal microbiota, while the combined therapy supplemented with BSEF could protect and restore the intestinal microbiota.

## Introduction

Guidelines about *H*. *pylori* eradication have stated that *H*. *pylori* infection should be eradicated in duodenal ulcer (DU) patients by the standard triple therapy^1^. Of the DU patients who received standard anti-*H*. *pylori* treatment, 10% to 30% develop antibiotic-associated diarrhea (AAD) and stool changes^2^. The reasons may relate to intestinal microbiota disorders caused by the use of anti-bacterial drugs^3,4^. Recent studies have reported that eradication of *H*. *pylori* caused perturbation of the gut microbiome and may indirectly affect the health of human^5^. However, the intestinal microbiota in DU patients is unknown. The effect on intestinal fungi caused by anti- *H*. *pylori* therapy, as well as a combined therapy with probiotic treatment in DU patients remains unclear. We hypothesized that (1) the intestinal microbiota in DU is different from that in healthy people; (2) *H*. *pylori* standard treatment disrupts intestinal microbiota; and (3) combined *Bacillus subtilis* and *Enterococcus faecium*(BSEF) treatment may improve the rate of *H*. *pylori* eradication, reduce side effects, and protect intestinal microbiota.

## Methods

### Study population

Healthy adults aged 18–65 years old were first screened to assess study eligibility. The exclusion criteria for the study were diabetes, hyper- or hypothyroidism, prior gastric or bariatric surgery, prior documented treatment for *H*. *pylori*, antibiotic or probiotics use within 4 weeks of enrollment, use of steroids or other immunomodulating drugs within 4 weeks of enrollment, recent vaccination and Charlson weighed comorbidity index <2. Written informed consent was obtained from qualified volunteers prior to study participation. Authors had access to the study data and reviewed and approved the final manuscript. The study protocol was reviewed and approved by the Medical Ethics Committee at the Chinese PLA General Hospital (PLAGH) (date of first registration:1/4/2013,registration number:S2013-051-01) and the Chinese Clinical Trial Registry (registration number:ChiCTR-TRC-13003228) on 19April 2013. We confirm that all methods were performed in accordance with the relevant guideline.

Each participant was asked to fill in 2 side-effect questionnaires to record the side effects according to severity: mild, moderate or severe^6,7^. The patients were also asked to record the improvement of symptoms and the status of ulcer healing under endoscopy before and after treatment^8^.

### Sample collection

The active DU patients were diagnosed in the Chinese PLA General Hospital (PLAGH) from 2013 to 2016. Urea breathing test (UBT) was performed in all patients. Biopsy specimens were taken from the antrum and corpus for a rapid urease test (RUT) and histology. *H*. *pylori* was considered present if two of the three were positive. When taking a gastroscope, take 1–2 ml of gastric juice, immerse the Acilit® pH test strip into the measured gastric juice, and read the pH of the gastric juice.

After the patients met the eligibility criteria and provided informed consent, we subsequently randomized patients using concealed allocation based on a list of random numbers, which was computer-generated. Forty *H. pylori* infected patients with active DU were randomly divided into a triple treatment group (TT, n = 20) and a triple therapy plus probiotic treatment group (TP, n = 20). In addition, 20 normal healthy persons were included as health check controls (HC, n = 20). Healthy persons meas no digestive diseases and other systemic diseases, and have not taken any drugs in the last month. Patients in the TT group received 2 weeks of standard triple therapy (20 mg esomeprazole, 500 mg clarithromycin, 1000 mg amoxicillin). Patients in the TP group received PCA followed by 6 weeks of the probiotics *Bacillus Subtilis* and coated capsules BSEF 500 mg 3 times daily. Follow-up endoscopy was conducted for all patients after stopping PCA for 4 weeks to check ulcer healing and *H*. *pylori* status. The volunteers were subsequently followed up at 2, 4, and 8 weeks post-*H*. *pylori* eradication. Stool samples (group TT fecal samples, TF; group TP fecal samples, PF) were collected pre-therapy and during each visit, and were frozen immediately at −80 °C until DNA extraction. TF1, TF2 and TF3 represent fecal samples of TT group for 2, 4 and 6 weeks of anti-Hp treatment, respectively. PF1, PF2 and PF3 represent fecal samples of TP group for 2, 4 and 6 weeks of anti-Hp treatment, respectively.

### Gene amplification and sequencing

The gut microbial genomic DNA was extracted from stool samples using a stool DNA extraction kit (QIAamp DNA Stool Mini Kit; cat. no. 51504). The primers of the bacterial 16S rDNA were as follows: The upstream primer was 5′-GTGCCAGCMGCCGCGGTAA-3′, and the downstream primer was 5′-GGACTACHVGGGTWTCTAAT-3′. The V4 region of stool bacterial 16S rDNA and the ITS region of fungi were amplified with a high-fidelity enzyme. The ITS1 region primer is ITS1-5F–ITS2; the ITS2 region primer is: ITS2-3F–ITS2- 4R. The library was constructed using New England Biolabs’ NEB Next® UltraTM DNA Library Prep Kit for Illumina library. Sequencing was completed using a Paired-End (PE) approach on the Illumina MiSeq high-throughput sequencing platform.

### Bioinformatics analysis

Quality control of the reads was performed by the QIIME software package to pick the high-quality reads that met the requirement^9,10^. Sequences with similarity greater than 97% were picked up using the operation command of the QIIME quality controller and were clustered into an operational taxonomic unit (OTU)^11^. The Ribosomal Database Project (RDP) software package was used for the homology alignment, as well as the species and genera taxonomic identification of the longest 16S rDNA sequence fragment of OTUs^12^. The database referred to the Greenbank at http://greengenes.lbl.gov/cgi-bin/nph-index.cgi. The resulting UniFrac distance matrices were used to perform Principal Coordinate Analysis (PCoA) to determine the similarity between groups of samples/time-points.

### Statistical methods

The raw data of the taxonomy summary results was exported to SPSS software version 20.0 (SPSS Inc., Chicago, IL) for statistical analysis. The mean abundance in percentage (%) and the 95% confidence interval (95% CI) for the phyla of stool microbiome at different time-points were calculated. Parametric paired-samples t-test was performed to compare the genera of the stool microbiome. Pearson’s Correlation Coefficient was also calculated to investigate across different time points; a two-tailed *P-*value of < 0.05 was considered significant.

### Compliance with ethical standards

The study protocol was reviewed and approved by the Medical Ethics Committee at the Chinese PLA General Hospital (PLAGH) (date of first registration:1/4/2013,registration numberS2013-051-01) and the Chinese Clinical Trial Registry (date of first registration:19/4/2013,registration number:ChiCTR-TRC-13003228).

## Results

### Clinical information

There was no significant difference in gender, age, and BMI among the 3 groups (*P* < 0.05), suggesting the comparability of the groups (Table 1).

**Table 1:** Basic information of the subjects.

Basic information	TT group	TP group	HC group	*P-*value
Number of cases	20	20	20	
**Gender**				
Male	13	12	12	0.9319
Female	7	8	8	
Ages	45.5 ± 8.4	45.2 ± 11.6	42.9 ± 12.3	0.2386
BMI	24.1 ± 2.6	24.4 ± 2.7	24.5 ± 3.0	0.8112

#### *H*. *pylori* eradication rate

Gastroscopy revealed that the ulcer healing rate of the TT group (90%) was lower than that of the TP group (100%), but there was no statistically significant difference (*P* = 0.4682).

A total of 16 cases (80%) in the TT group were successfully eradicated, and 17 cases (85%) were successfully eradicated in the TP group. No difference in the success rate of the *H*. *pylori* eradication was observed between the 2 groups (*P* > 0.05).

#### Gastric pH value

**Figure 1:**
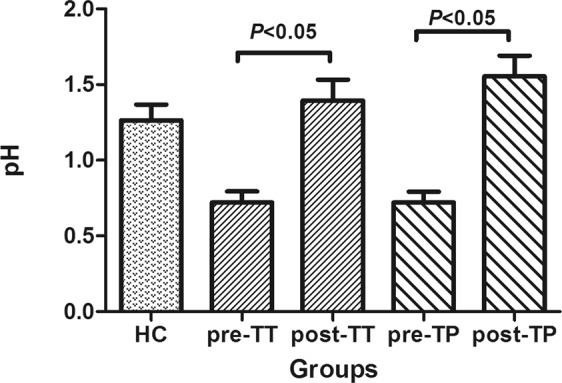
Gastric pH value before and after treatment.

The gastric pH values of DU patients in the pre-TT and the pre-TP groups were significantly different from that in the HC group (*P* < 0.01), while the TT and the TP groups after treatment (post-TT and post-TP groups) showed no difference with the HC group (*P* = 0.274). Significant differences were observed between the pre-TT and the post-TT group (*P* < 0.01), as well as between the pre-TP and post-TP groups (*P* < 0.01) (Table 2 and Fig. 1).

**Table 2:** pH value of gastric juice before and at 4w after treatment (Mean ± SD).

Group	Number of cases	pH value Before treatment	pH value After treatment	*P-*value
TT Group	20	0.720 ± 0.330	1.395 ± 0.613	0.0001*
TP Group	20	0.720 ± 0.321	1.555 ± 0.610	0.0000*
HC Group	20	1.265 ± 0.456

#### Side effects

There were more side effects in the TT group than in the TP group (35% vs 20%). The most common side effects were diarrhea (35%), followed by nausea (30%), abdominal pain (25%), abnormal taste (20%), abdominal bloating (15%), anorexia (10%), and constipation (5%). These side effects mostly occurred during treatment, and were predominantly mild to moderate, whereas all the cases of the TP group were mild.

### Fecal microbiome in DU patients

The average Raw PE number obtained for each bacterial sample was 65630 and fungi sample was 54549. The dilution curve was drawn by 3 methods, including the OTU numbers, the Chao1 and the Shannon 3 for mutual validation, and the results suggested a trend of reaching gentleness at 10,000 sequences.

#### Bacterial microbiome

**Figure 2:**
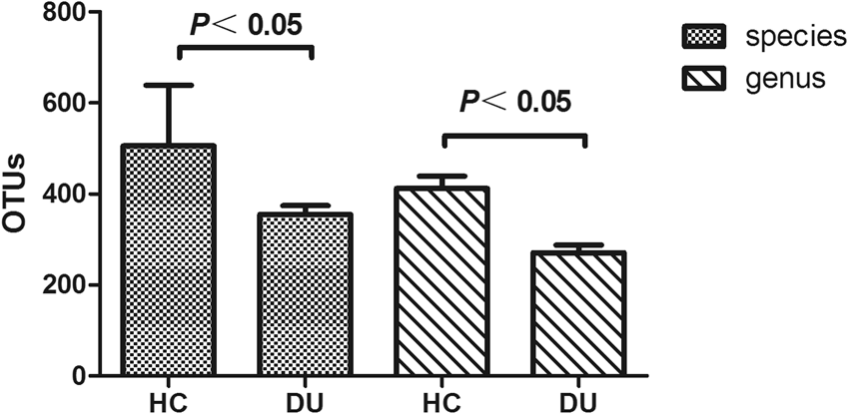
Comparison of OTUs from the 2 groups at the species and genus levels.

OTU analysis: Statistical analysis showed that the OTU number was lower in the DU group compared with that in the HC group, either at the 97% OTU species-level (HC group 505.33 ± 133.63 vs DU group 355.07 ± 104.11 *P* = 0.0003) or at the 95% OTU genus level (HC group 412.17 ± 114.75 vs DU group 271.28 ± 89.73, *P* = 0.0001). Differences between the 2 groups were statistically significant (Fig. 2).

Annotation of species and analysis of differential bacteria: The statistical analysis revealed that the bacterial flora community was derived from 38 bacterial phyla, 110 bacterial classes, 157 bacterial orders, 230 bacterial families, and 275 bacterial genera.

At the phylum level, the abundance distribution showed that in the HC group, the dominant phyla were *Firmicutes*, *Bacteroidetes*, and *Proteobacteria*. These 3 bacterial phyla were abundant in every sample with high percentages, accounting for 45.5%, 33.0%, and 13.2%, respectively, and together accounting for 91.7% of the total bacteria. In the DU group, the dominant phyla were also *Firmicutes*, *Bacteroidetes*, and *Proteobacteria*, accounting for 52.5%, 25.7%, and 15.7%, respectively, and together accounting for 93.9% of the total bacteria. Although the percentages of the 3 bacterial phyla have changed, the differences between the DU group and the HC group were not statistically different. Comparing the other 35 bacterial phyla between the DU group and the HC group revealed a total of 7 bacterial phyla with statistically significant differences: *Actinobacteria*, *Gemmatimonadetes*, *Nitrospirae*, *Chlorobi*, *Thermi*, *WS3*, and *Caldithrix*. The percentages of these 7 bacterial phyla were all lower in the DU group compared with those in the HC group.

At the genus level, the dominant genera in the HC group were *Bacteroides* (22.8%), *Faecalibacterium* (13.6%), *Prevotella* (5.3%), *Roseburia* (5.1%), *Ruminococcus* (4.5%), *Escherichia* (4.2%), *Bifidobacterium* (4.2%), *Lachnospira* (2.3%), etc.; the dominant genera in the DU group were *Bacteroides* (17.1%), *Faecalibacterium* (15.3%), *Escherichia* (7.8%), *Ruminococcus* (5.5%), *Roseburia* (4.7%), *Prevotella* (4.6%), *Akkermansia* (2.9%), *Parabacteroides* (2.4%), *Bifidobacterium* (2.0%), etc. There were a total of 55 differential bacteria at the genus level, 54 of which were less in the DU group than those in the HC group, except the *Natronomonas* genus that increased in the DU group.

**Figure 3:**
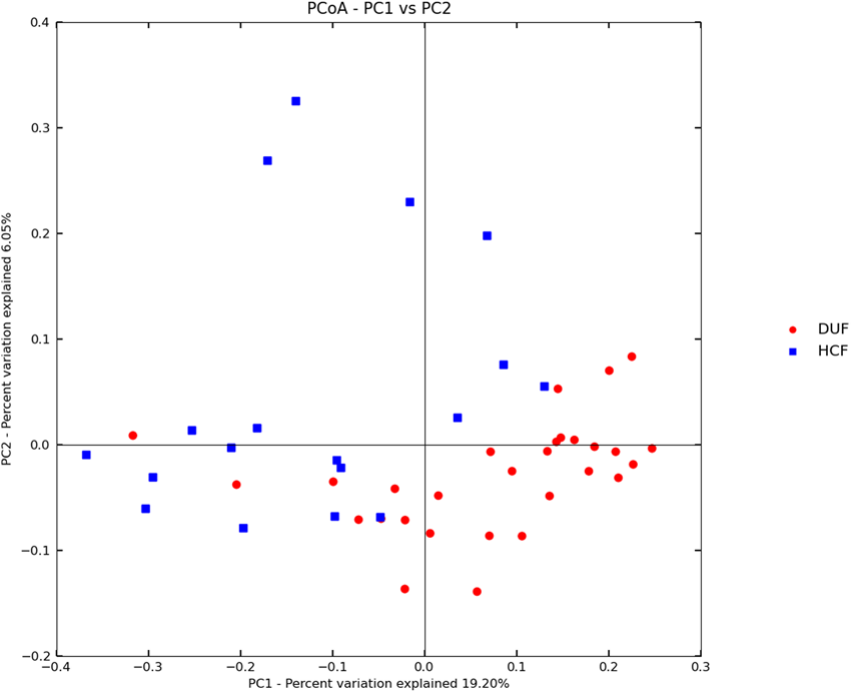
PCoA analysis (blue denotes the HC group and red denotes the DU group).

Analysis of species composition: The PCoA analysis found that the intra-group distances between each sample within the HC group and the DU group were smaller than the inter-group distances, suggesting the similarity of the species compositions (Fig. 3).

**Figure 4:**
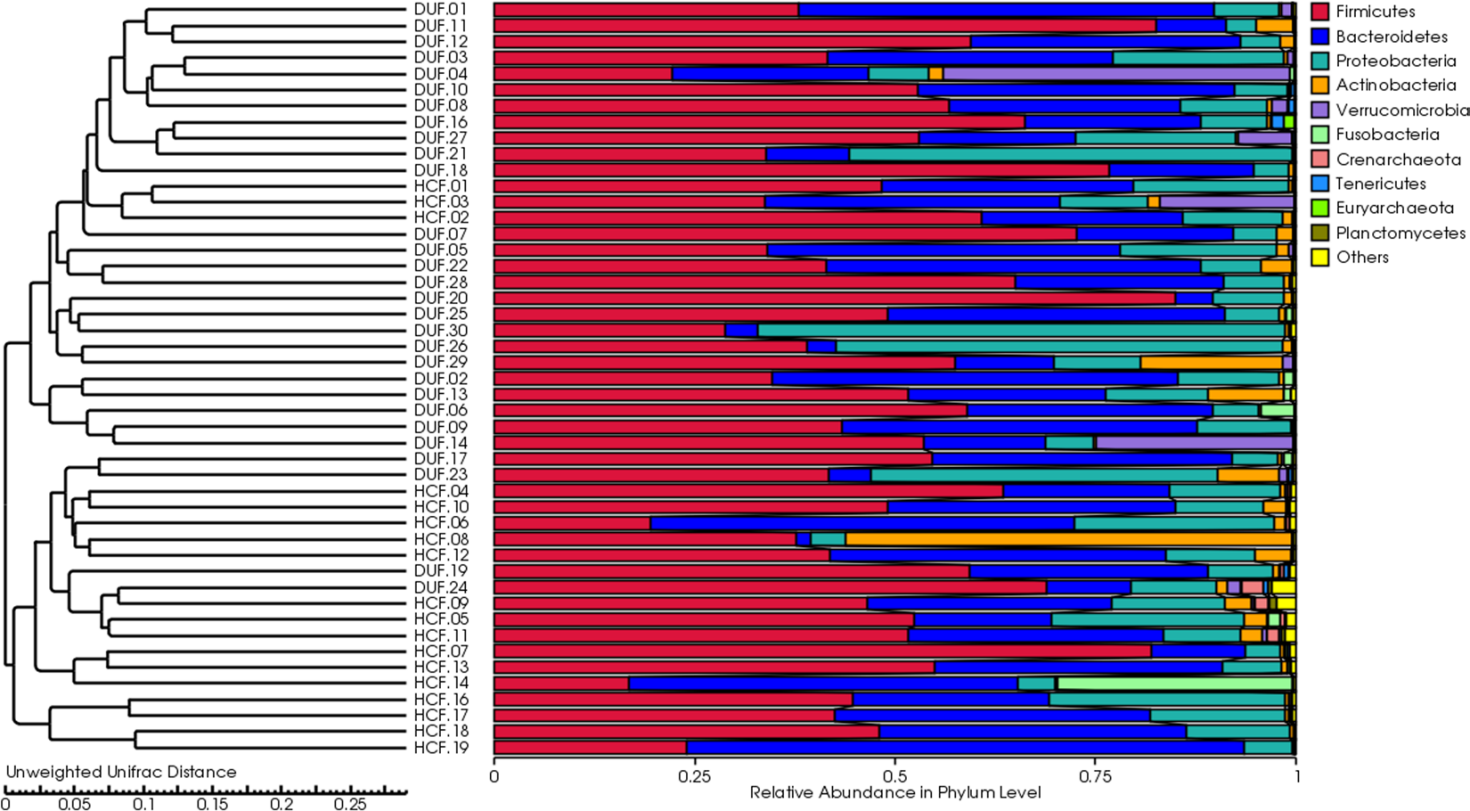
Unweighted UniFra distance clustering tree of samples.

The UPGMA (Unweighted Pair Group Method with Arithmetic Mean) method was used to input the unweighted UniFrac clustering matrix, which performed cluster analysis on samples. In the cluster diagram (Fig. 4), we found that the bacterial flora structure among all the samples exhibited both clustering and various degrees of overlapping. However, only a few samples could be clustered between the DU group and the HC group, with a general trend of high similarity between samples within groups. Therefore, the DU group or the HC group was considered as a homologous group, and samples from such groups exhibited internal clustering.

### Fungi microbiome

#### Fungi microbiome

**Figure 5:**
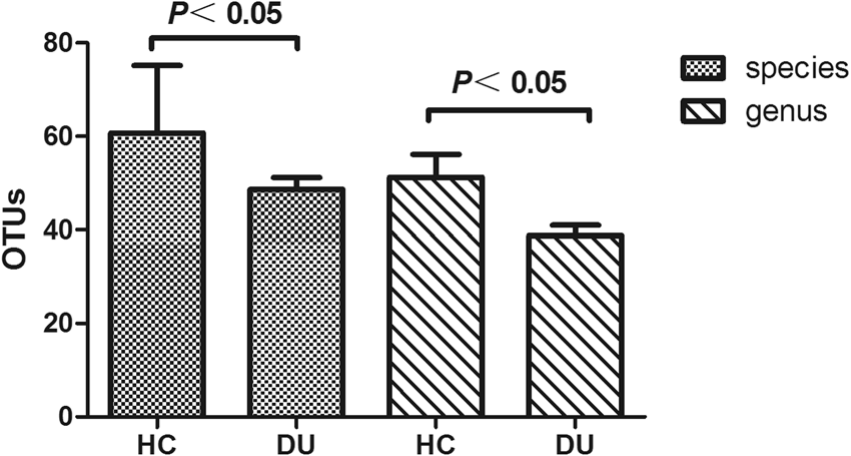
Comparison of OTUs of the 2 groups of at the species and genus level.

OTU analysis: The OTU number was lower in the DU group compared with that in the HC group, either at the 97% OTU species level (HC group 60.70 ± 14.46 vs DU group 48.65 ± 10.26*P* = 0.0180) or at the 95% OTU genus level (HC group 51.20 ± 15.49 vs DU group 38.77 ± 9.13*P* = 0.0141). Differences between the 2 groups were statistically significant (Fig. 5).

Annotation of species and analysis of differential fungi: According to the annotation results of species, most of the fungal communities came from 2 fungal phyla, 31 fungal classes, 53 fungal orders, 90 fungal families, and 104 fungal genera.

At the phylum level, the abundance distribution showed that the abundance of *Ascomycota* was 59.6% in the HC group and 58.8% in the DU group; the abundance of *Basidiomycota* was 37.0% in the HC group and 40.3% in the DU group. There was no difference in the fungal phyla between the 2 groups.

At the genus level, the abundance distribution showed that in the HC group the dominant genera with the fungal flora abundance of more than 1% were *Sarcinomyces* (49.2%), *Malassezia* (33.1%), *Alternaria* (5.9%), *Pseudozyma* (2.4%), *Simplicillium* (1.9%), and *Aspergillus* (1.5%); in the DU group, the dominant genera with the fungal flora abundance of more than 1% were *Sarcinomyces* (53.9%), *Malassezia* (35.8%), *Pseudozyma* (3.6%), *Simplicillium* (2.2%), and *Aspergillus* (1.0%). There were 2 differential fungi at the genus level: *Pseudozyma* significantly increased in DU (3.6%) compared with that in HC (2.4%) (*P* = 0.01499), and *Candida* significantly decreased in DU (0.001%) compared with that in HC (0.024%) (*P = *0.02113).

**Figure 6:**
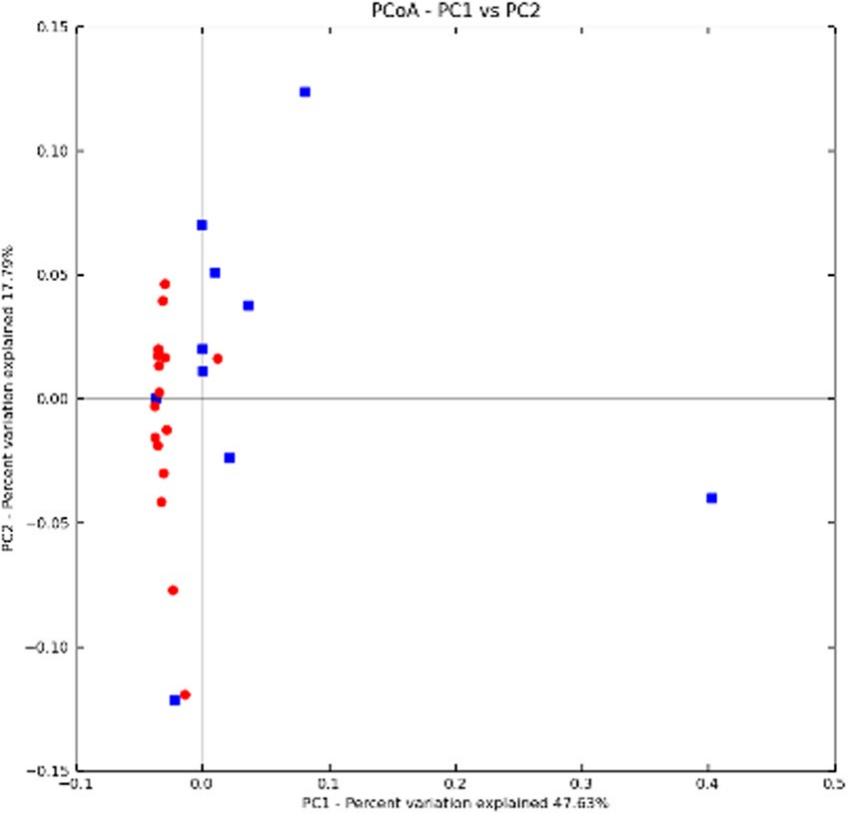
PCoA analysis (blue represents the HC group, and red represents the DU group).

Analysis of species composition: PCoA analysis was performed, and the results are shown in Fig. 6. It can be seen that the majority of the samples from the DU groups were clustered in the lower left quadrant, whereas samples from the HC group tended to locate in the upper part of the quadrant. These results suggest that the fungal flora compositions between the DU group and the HC group are not exactly the same, while the internal fungal flora structures within groups are more similar.

**Figure 7:**
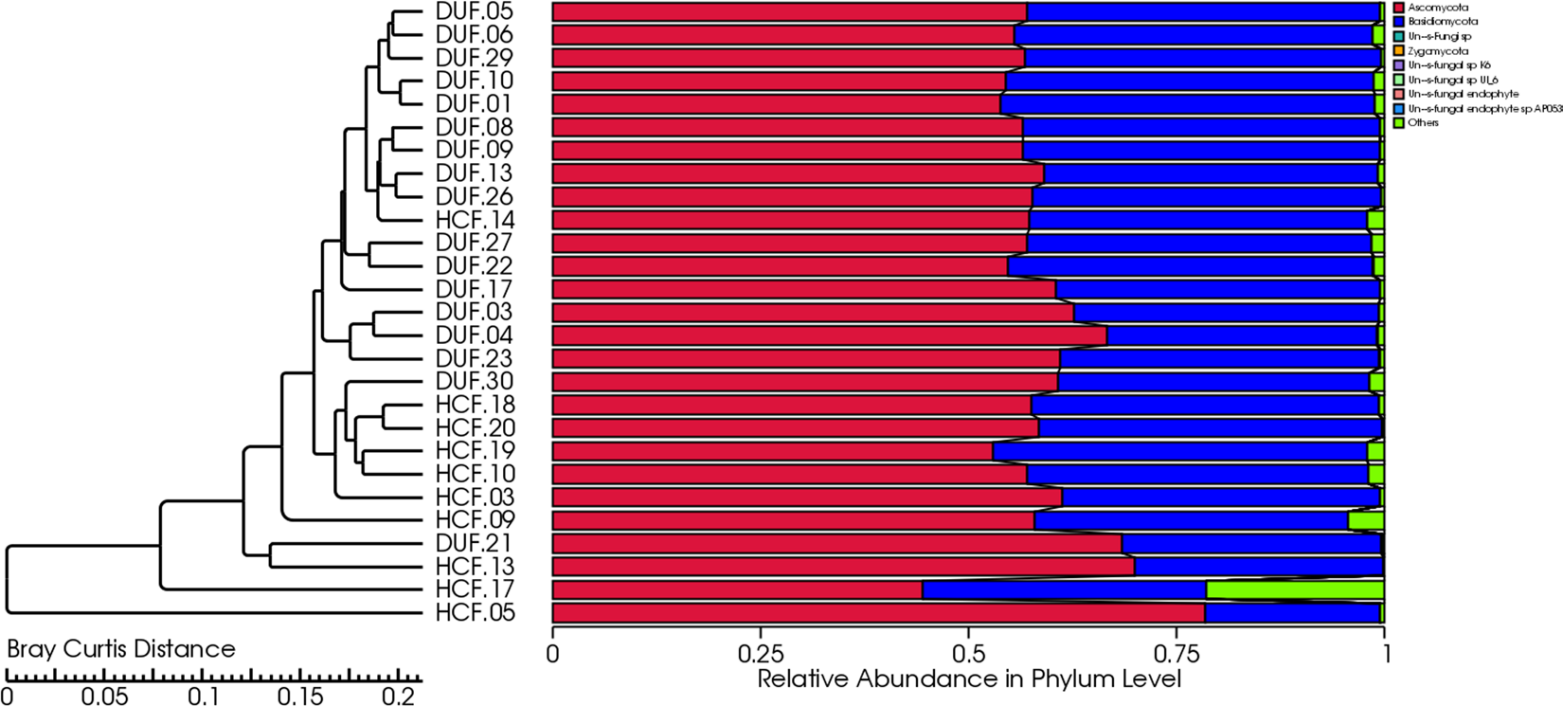
Clustering tree.

Next, samples were subjected to cluster analysis (Fig. 7). In the clustering diagram, we found that the fungal flora structure among all the samples exhibited both clustering and various degrees of overlapping. However, only a few samples could be clustered between the DU group and the HC group. The general trend was that samples from the DU group or the HC group were first clustered, suggesting that either the DU group or the HC group had a high intra-group similarity.

### Changes of intestinal stool microbiome after *anti-H*. *pylori* treatment

#### Bacterial microbiome

**Figure 8:**
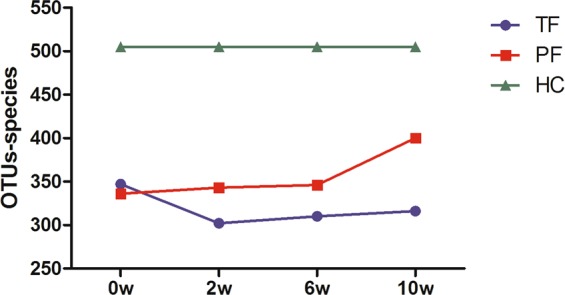
OTUs at different time points before and after treatment.

OTU analysis: Statistical analysis of OTUs at the bacterial species level was carried out for 4 different time points in the TT group and the TP group (Table 3 and Fig. 8); 0 week (w) represents the DU group before treatment (i.e., with ulcers). Intra-group comparisons were first performed within both groups, and we found that, except in the TT group where differences were observed between DU and TF1 (*P* = 0.03159) and between DU and TF2 (*P* = 0.04526), there was no statistical difference in the rest of the pairwise comparisons. In other words, at the end of the anti-*H*. *pylori* treatment and at 6w after treatment, the intestinal bacterial flora abundance in the TT group decreased compared with that in the untreated group, whereas the TP group showed no significant changes compared with the untreated group.

**Table 3:** OTUs at different time points in the two groups.

	TT Group	TP Group
0w	347.41 ± 82.61	335.66 ± 85.43
2w	302.40 ± 48.11*	342.67 ± 68.58#
6w	310.07 ± 34.14*	346.07 ± 63.33#
10w	316.40 ± 48.02	400.00 ± 141.34#

(*denotes *P* < 0.05 compared with 0w, and # denotes *P* < 0.05 when comparing the 2 groups at the same time points).

When the 2 groups were compared with the HC group before and after treatment at different time points, respectively, most of the compared groups showed statistical differences, except between the TP group and the HC group (Table 4).

**Table 4:** *P-*values from the inter-group comparisons of the OTUs at different time points.

Group	HC	PF1	PF2	PF3
HC	—	0.00068**	0.00043**	0.11850
TF1	0.00000**	0.02077*	—	—
TF2	0.00000**	—	0.01052*	—
TF3	0.00001**	—	—	0.03916*

(*denotes *P* < 0.05, ** denotes *P* < 0.01).

Annotation of species and analysis of differential bacteria:

1. The intestinal bacterial changes between groups at the same time points. At the end of the 2w treatment, there was no significant change in intestinal bacterial flora at the phylum level between the 2 groups, whereas at the genus level, a total of 23 differential bacterial genera (*P* < 0.05) were discovered. After 6w of treatment, there were 28 differential bacterial genera, and at 10w, there were 11. Details of the differential bacterial genera are listed in the following table (Table 5).

**Table 5:** Differential bacterial genera between the 2 groups at different time points.

	2w	6w	10w
The bacterial genera higher in the TP group than the TT group	*Actinomadura*	*Actinomadura*	*Achromobacter*
	*Atopobium*	*Anaerofilum*	*Actinomyces*
	*Brevundimonas*	*Candidatus Nitrososphaera*	*Bradyrhizobium*
	*Butyrivibrio*	*Candidatus Solibacter*	*Candidatus Solibacter*
	*Coprococcus*	*Coprococcus*	*Coprococcus*
	*Coraliomargarita*	*Coraliomargarita*	*Chelativorans*
	*Corynebacterium*	*Dechloromonas*	*Cetobacterium*
	*Desulfobacca*	*Desulfomonile*	*Cupriavidus*
	*Desulfobulbus*	*Desulfobulbus*	
	*Eggerthella*	*Dok59*	
	*Faecalibacterium*	*Dorea*	*Thiobacillus*
	*GOUTA19*	*Leuconostoc*	*Leuconostoc*
	*Helicobacter*	*Luteimonas*	
	*Lewinella*	*Lewinella*	
		*Luteolibacter*	
		*Neisseria*	
		*Nitrosopumilus*	
	*Oscillospira*	*Oscillospira*	
	*Rhodobacter*	*Parapedobacter*	
	*Rhodoplanes*	*Planctomyces*	
	*Roseburia*	*Thauera*	
	*Roseomonas*	*Syntrophobacter*	
	*Rubrivivax*	*Syntrophomonas*	
	*Thauera*	*T78*	
		*Thermomonas*	
	*Thiobacillus*	*Thiobacillus*	
The bacterial genera lower in the TP group than the TT group	*Dialister*	*Dialister*	*Dialister*
		*Plesiomonas*
2. The intestinal bacterial changes within groups at different time points. In the TT group, among the top ten bacterial phyla at the phylum level, pairwise comparisons within the group showed that differences were only observed in the *Bacteroidetes* phylum and the *Tenericutes* phylum: the relative abundance of the *Bacteroidetes* phylum increased in TF2 compared with that in DU (*P* = 0.03664), and the relative abundance of the *Tenericutes* phylum decreased in TF1 compared with that in DU (*P* = 0.01807). There was no statistical difference in changes of different bacterial phyla at the remaining time points (Fig. 9).

**Figure 9:**
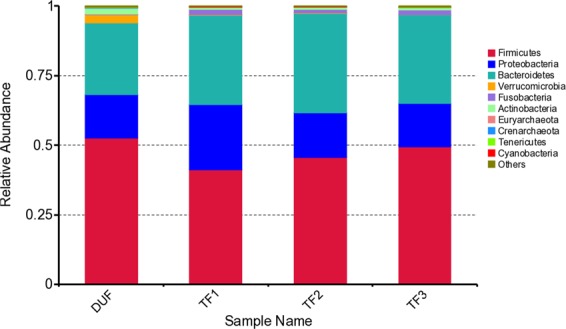
Abundance changes of bacterial phyla in the TT group before and after treatment.

**Figure 10:**
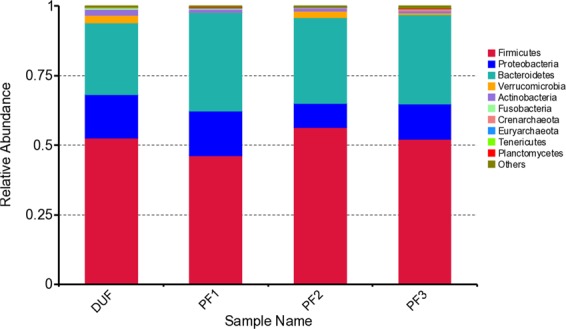
Abundance changes of bacterial phyla in the TP group before and after treatment.

In the TP group, among the top 10 bacterial phyla at phylum level, pairwise comparisons within the group showed that differences were only observed in the *Firmicutes* phylum, the relative abundance of which increased in PF2 compared with that in PF1 (*P* = 0.01356). There was no statistical difference in changes of different bacterial phyla at the remaining time points (Fig. 10).

At the genus level, among the 4 different time points in the TT group, pairwise comparisons within the group revealed a total of 8 significantly different (*P* < 0.05) bacterial genera, the bacterial flora abundance of which was more than 0.1% of the total samples.

At the genus level, among the 4 different time points in the TP group, pairwise comparisons within the group revealed a total of 5 significantly different (*P* < 0.05) bacterial genera, the bacterial flora abundance of which was more than 0.1% of the total samples. Such differential bacterial genera were fewer in the TP group than that in the TT group, and their abundance percentage was very low. These results suggest that the changes of bacteria at different time points in the TP group were slight, and were from only a small number of bacteria. These bacterial changes were stable, and exhibited a trend of gradual transition. In the TT group, however, the bacterial changes were larger, came from a larger number of bacteria, and presented more intense amplitudes.

#### Fungi microbiome

**Figure 11:**
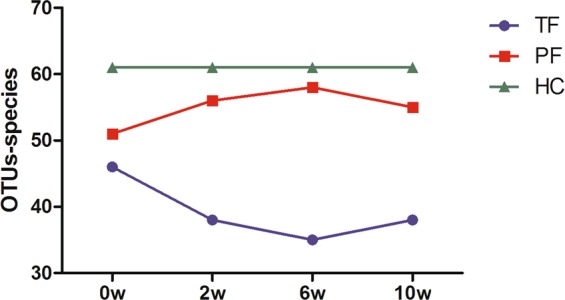
OTUs at different time points before and after treatment.

OTU analysis: The results revealed that at the 3 time points after TT treatment, the number of OTUs of the intestinal fungal flora decreased compared with that in DU, and was statistically different (*P* < 0.05); before and after TP treatment, however, there was no obvious change in the number of OTUs. There were differences between the 2 treatment approaches at the same time points (Table 6 and Fig. 11).

**Table 6:** OTUs at different time points in the two groups.

	TT Group	TP Group
0w	46.12 ± 9.44	51.00 ± 10.38
2w	37.71 ± 8.03*	55.62 ± 6.69#
6w	35.30 ± 5.50*	58.14 ± 6.20#
10w	37.62 ± 6.02*	55.25 ± 10.27#

(*denotes *P* < 0.05 compared with 0w, and # denotes *P* < 0.05 when comparing the 2 groups at the same time points).

When the 2 groups were compared with the HC group before and after treatment at different time points, respectively (Table 7), it could be seen that there was no statistical difference among the 3 time points after TP treatment and the HC group, whereas there were statistically significant differences among the 3 time points in the TT group and the HC group.

**Table 7:** *P* values from the inter-group comparisons of the OTUs at different time points.

Group	HC	PF1	PF2	PF3
HC		0.28750	0.40350	0.2505
TF1	0.00442**	0.00021**		
TF2	0.00060**		0.00043**	
TF3	0.00318**			0.01979*

(*denotes *P* < 0.05, ** denotes *P* < 0.01).

Annotation of species and analysis of differential fungi:

1. The intestinal fungal changes between groups at the same time points. At the phylum level, the 2 treatment approaches showed no obvious difference in the intestinal fungal flora at any of the 3 time points after treatment. At the genus level, some of the fungi were significantly different (*P* < 0.05): at the end of 2w treatment, a total of 5 differential fungal genera were observed. Details of the differential fungal genera are listed in the following table (Table 8).

**Table 8:** Differential fungal genera between the two groups at different time points.

	2w	6w	10w
The fungal genera higher in the TP group than the TT group	*Aspergillus*	*Trichoderma*	*Aureobasidium*
The fungal genera lower in the TP group than the TT group	*Cladosporium*	*Cladosporium*	*Cladosporium*
	*Pseudozyma*	*Pseudozyma*	*Pseudozyma*
	*Simplicillium*	*Simplicillium*	*Simplicillium*
		*Sarcinomyces*	*Sarcinomyces*
	*Fusarium*		*Fusarium*
			*Candida*
2. Intestinal fungal changes within groups at different time points At the phylum level, there was no difference among the 4 time points either in the TP group or in the TT group. At the genus level, among the 4 time points in the TT group, pairwise comparisons within the group revealed that only the *Pseudozyma* genus and the *Cladosporium* genus showed significant differences (*P* < 0.05), and the trends of the changes at the 4 time points are shown in the figure below; among the 4 time points in the TP group, pairwise comparisons within the group revealed a total of 6 differential fungal genera (*P* < 0.05), more than those in the TT group. Moreover, the relative abundance of the differential fungal genera was higher than that in the TT group, suggesting that the fungal changes at different time points in the TT group were slight and came from only a small number of fungi, whereas the fungal changes in the TP group were larger and came from a larger number of fungi.

## Discussion

Eradication of *H*. *pylori* is essential for the treatment of the *H*. *pylori-*associated DU^2^. PCA therapy for *H*. *pylori* eradication has been in clinical use for a long time. Due to the increased rates of drug resistance and side effects, most studies have shown that the addition of probiotics reduces side effects, but whether these probiotics additives improve the eradication rate is still controversial^6,13–16^. Chen *et al*.^17^ found that *Lactobacillus delbrueckii* and *Lactobacillus plantarum*-18 additives had significant anti-*H*. *pylori* effects. The anti-*H*. *pylori* effects of the 2 strains mainly related to the pH value of the cells’ supernatant and their metabolites.

However, some researchers have questioned the methods that combine anti-*H*. *pylori* therapy with probiotics additives^18^. But the efficacy of the therapy can be increased if acid-resistant and bile salts-resistant *BSEF* preparations are added. Park *et al*.^19^ have shown that the therapy combining 2 live bacteria-*BSEF* improved eradication rates and reduced side effects. Results from our study showed that the combination therapy with *BSEF* did not significantly improve eradication rates but reduced the incidence of side effects.

The application of PPIs in triple therapy increased the pH value of gastric juice, resulting in a low acidic environment of the stomach. The low acidic environment weakens the bacteria killing effect of the therapy, and may change intestinal microecology^20^. Our study found that the pH value of the gastric juice in DU patients (0.72) was significantly lower than that in normal people (1.27). The pH value returned to normal after anti-*H*. *pylori* treatment. We have investigated, for the first time, the intestinal bacterial and fungal flora in DU, and found that the diversity and abundance of intestinal bacteria and fungi were significantly lower than those in healthy people.

The presence of human intestinal microbiota is vital for the health of the host. These microorganisms play a unique role in human nutrition, growth, metabolism, resistance to pathogens, immune regulation, and the like^21–23^. However, the use of antibiotics may break the steady state of the interaction between the microorganisms and the host^24,25^. Meanwhile, the impact of PPIs on intestinal microbiota has also been of concern^26,27^. PPIs weaken the role of gastric acid in eliminating exogenous bacteria, leading to easy invasion and colonization of exogenous pathogenic bacteria of the human body. A number of studies have confirmed that when receiving the combined application of PPIs and antibiotics for *H*. *pylori* infection, 10% to 30% of the patients experienced changes in defecation frequency and stool characteristics^28–32^. The reasons for this may relate to intestinal microbiota disturbance. This was also confirmed in our study, which has revealed that the incidence of diarrhea was as high as 35% during triple therapy.

Oh *et al*.^28^ showed that probiotic supplementation can reduce the antibiotic-induced alteration and imbalance of the intestinal microbiota composition. Despite the changes in intestinal microbiota, this study suggested that the impact of triple therapy on the intestinal microbiota was short-term about 1 month. Our study found that anti-*H*. *pylori* treatment led to an imbalance of intestinal homeostasis and the reduction of diversity, especially during the triple only treatment. After combination therapy with probiotics additives, changes of intestinal microbiota were more moderate: intestinal bacterial diversity at the 10^th^ week was not significantly different from that in healthy people, while intestinal fungal diversity has always remained similar to that in the HC group.

Studies based on traditional microscopy have revealed a high incidence of fungal infection in patients with gastric ulcer, gastric cancer and gastric remnants^33,34^. So far, there is no report on sequencing results of intestinal microbiota in DU patients. Our study applied a high-throughput sequencing method, for the first time, to sequence intestinal fungal colonies in DU patients. The annotation of species found that the fungal communities were from 2 fungal phyla, 31 fungal classes, 53 fungal orders, 90 fungal families, and 104 fungal genera. In the HC group, the dominant genera were different with the DU group. The probiotic drugs used in our study contain 2 important strains of bacteria that promote the health of human intestines. In addition to the contributions from the bacteria themselves, these strains also promote the growth of other bacteria, especially thick-walled bacteria, such as *Bifidobacterium*. In the probiotic preparation we used, *E*. *faecium* is a gram-positive and facultative anaerobic bacterium. This bacterium reproduces rapidly, and has a strong inhibitory effect on pathogenic bacteria; *B*. *subtilis* is a gram-positive and aerobic bacterium, which consumes intestinal oxygen upon its intestinal colonization, resulting in biological oxygen deprivation. Such biological oxygen deprivation leads to a reduction in local oxygen concentration, creating an appropriate environment for the growth of normal intestinal anaerobic microbiota. This promotes the growth of *Bifidobacterium*, *Lactobacillus*, enterococci, and other beneficial bacteria, and inhibits the growth of *Escherichia coli*, dysentery bacili, *Staphylococcus aureus*, and other pathogenic bacteria, thereby restoring the balance of intestinal microbiota^33^. Studies have also found that *B*. *subtilis* produces a variety of digestive enzymes, further breaking down protein, fat, carbohydrates, and fibrin, etc. *B*. *subtilis* also produces lysozyme and more than 80 types of antibacterial compounds, thereby inhibiting the colonization and growth of pathogenic bacteria^34^. Moreover, *B*. *subtilis* inhibits the activity of all the *H*. *pylori* microbeads by secreting amicoumacin A and non-amicoumacin antibiotics. This inhibitory effect is not subject to acid restriction and is resistant to proteolytic cleavage. Based on the aforementioned mechanisms, BSEF formulations can be used against the intestinal microbiota imbalance caused by triple therapy.

## Data Availability

The datasets generated and/or analysed during the current study are available from the corresponding author and first author on resonable request.
